# Acute care surgery model in the COVID-19 pandemic era

**DOI:** 10.3205/dgkh000442

**Published:** 2023-08-04

**Authors:** Nader Aghakhani, Bahman Alinezhad, Mehdi Azami, Saied Amini Rarani

**Affiliations:** 1 Food and Beverages Safety Research Center, Urmia University of Medical Sciences, Urmia, Iran; 2 Department of General Surgery, School of Medicine Imam Khomeini Hospital, Urmia University of Medical Sciences, Urmia, Iran; 3 Skin Diseases and Leishmaniosis Research Center, Isfahan University of Medical Sciences, Isfahan, Iran; 4 Department of Medical Parasitology and Microbiology, Hojjatieh Medical Diagnostic Laboratory, Hojjatieh Hospital, Isfahan, Iran; 5 Basir Laboratory Research and Development Center, Basir Medical Diagnostic Laboratory, Isfahan, Iran; 6 Department of Operating Room, Nursing and Midwifery Care Research Center, Isfahan University of Medical Sciences, Isfahan, Iran

## Letter to the editor

Dear editor,

The COVID-19 global pandemic caused interruptions in the admission of patients to the emergency unit and a reduction in the incidence of optional surgeries, having a substantial effect on surgical practice.

On the other hand, the effects of COVID-19 on surgical professional problems, including the danger of intraoperative viral transmission, illness among health-care staff, and other influences on surgical training, have been discussed many times [1].

The current need for ventilators, staff, and space is a limiting factor for the deployment of surgical resources during the pandemic training, to the point where the provision of essential surgery is being compromised in multiple areas, such as operating rooms, the number of surgeons, and operating staff.Aroung the world, many people who are suffering from surgical complaints are affected both immediately and over time by the strain these limitations impose on the system [2].

Patients should be thoroughly observed after surgery, since there are signs that undetected COVID-19 may make recovery more complicated. Pulmonary complications following surgery are relatively unusual. Hence, it is crucial to take COVID-19 into account both before and after surgery [3].

Maintaining open pathways for routine diagnosis and treatment requires continuing institutional efforts, particularly in terms of building within-hospital networks for “contaminated” and “clean” patient flow. The length of the acute phase of the pandemic and the amount of time required to resume routine surgical procedures are two important sources of unpredictability.

If any aspect of the surgical procedures we are planning today are suspended, there is considerable probability of a rebound effect when the COVID-19 catastrophe is finally over. Delaying time-sensitive ‘elective’ procedures, such as transplants or cancer surgery, may cause avoidable deaths, health problems, and a decline in quality of life [4].

No information is currently available on the quality of the impact of this loss of surgical ability on the surgical condition and related health of the patients, as well as their well-being, risk of disability, functional capacity, and adverse effects on prognosis.

An organized framework for appraising the delivery of surgical procedures during the COVID-19 pandemic should be considered. Thus, a comprehensive supportive program that includes analysis, forecasting, investigation, and communication for anesthesia and surgical facilities for upcoming pandemics is also required [5].

Hospitals and health-care settings have had to change their structures during the pandemic. Therefore, the need for increased intensive care capacity compelled the transformation of waiting rooms and even recovery facilities into ICUs. Outpatient health-center visits were canceled, carried out online or over the phone, while health-care professionals were redistributed/reassigned. 

These alterations undoubtedly had an impact on the surgery departments; elective surgeries were postponed, healthcare professionals were assigned to support the ICUs, and surgeons were moved into closed workplaces to prevent infections. Certainly, a decrease in acute care surgery activity (ACSA) was one of these modifications. Acute care surgery (ACS) can be an innovative surgical domain that includes emergency general surgery, trauma surgery, and surgical intensive care [6].

In conclusion, the COVID-19 pandemic has created a unique chance to assess the effectiveness of the ACS model during this disruptive era, challenging health care organizations and hospitals with an intensified demand for both material assets and health personell.

## Notes

### Competing interests

The authors declare that they have no competing interests.

### Author’s ORCID

The ORCID ID of Rarani SA is: 0000-0003-4700-0211
